# Novel mutation identified in severe early-onset tumor necrosis factor receptor-associated periodic syndrome: a case report

**DOI:** 10.1186/s12887-017-0856-2

**Published:** 2017-04-20

**Authors:** Suhas M. Radhakrishna, Amy Grimm, Lori Broderick

**Affiliations:** 1 Department of Pediatrics, Division of Allergy, Immunology, Rheumatology and Kawasaki Disease, University of California San Diego and Rady Children’s Hospital, 9500 Gilman Drive #0760, La Jolla, San Diego, CA 92093 USA; 2 Department of Pediatrics, University of California San Diego and Rady Children’s Hospital, San Diego, CA USA; 3 Allegro Pediatrics, Bellevue, WA USA

**Keywords:** Autoinflammatory disease, Periodic fever syndrome, Tumor necrosis factor receptor associated periodic syndrome, Kawasaki disease, Anakinra, Case report

## Abstract

**Background:**

Tumor Necrosis Factor Receptor-Associated Periodic Syndrome (TRAPS) is the second most common heritable autoinflammatory disease, typically presenting in pre-school aged children with fever episodes lasting 1–3 weeks. Systemic symptoms can include rash, myalgia, ocular inflammation, and serositis.

**Case presentation:**

Here we report an unusual presentation of TRAPS in a 7 month old girl who presented with only persistent fever. She was initially diagnosed with incomplete Kawasaki Disease and received IVIG and infliximab; however, her fevers quickly recurred. Subsequent testing revealed a urinary tract infection, but she did not improve despite appropriate therapy. As fever continued, she developed significant abdominal distension with imaging concerning for appendicitis, followed by hyperthermia and hemodynamic instability. Given her protracted clinical course and maternal history of a poorly defined inflammatory condition, an autoinflammatory disease was considered. Therapy with anakinra was initiated, resulting in rapid resolution of fever and normalization of inflammatory markers. She was found to have a previously unreported mutation, Thr90Pro, in the *TNFRSF1A* gene associated with TRAPS. This novel mutation was also confirmed in the patient’s mother and maternal uncle.

**Conclusions:**

This report reviews a severe case of TRAPS in infancy associated with a novel mutation, Thr90Pro, in the *TNFRSF1A* gene, and emphasizes that autoinflammatory disease should be considered in the differential of infants with fever of unknown origin.

**Electronic supplementary material:**

The online version of this article (doi:10.1186/s12887-017-0856-2) contains supplementary material, which is available to authorized users.

## Background

Autoinflammatory diseases, also known as periodic fever syndromes, are caused by dysregulation of the innate immune response with inappropriate activation of interleukin-1 and tumor necrosis factor pathways [1]. These differ from typical autoimmune disorders in that they are not mediated by autoantibodies and are often monogenically inherited. The diagnosis of an autoinflammatory disease should be considered in the setting of recurrent fever without a source. Family history of recurrent fevers or inflammatory symptoms may lend support to the diagnosis.

In Tumor Necrosis Factor Receptor-Associated Periodic Syndrome (TRAPS), the average age of onset is 3 years of age, but onset has been reported as early as 4 days [2] and up to the sixth decade of life. Flares, lasting from 5 days to several weeks, may occur spontaneously or in relation to a trigger such as a minor illness. Symptoms include fever, migrating myalgia, arthralgia or arthritis, rash, and serous membrane inflammation that manifests as chest and abdominal pain [1]. Arthralgia is more common than arthritis, and affects large joints as well as wrists and temporomandibular joints. Centrifugal migratory erythematous rashes are present in the majority of cases, but other patterns, including urticaria, are possible. Periorbital edema is also common and can be associated with uveitis or conjunctivitis. Secondary AA amyloidosis is the most severe long term complication of TRAPS, affecting 10–20% of untreated patients [3]. Morbidity and mortality associated with amyloidosis is generally due to nephrotic syndrome and renal failure [1], but other organ systems can be affected.

TRAPS is due to mutations in *TNFRSF1A* that encodes the Tumor Necrosis Factor (TNF)-∝ receptor, and is inherited in an autosomal dominant fashion. Mutations are primarily single nucleotide missense mutations in exons 2, 3, 4 and 6 that affect the three-dimensional structure of the receptor. Normally, binding of TNF-∝ to the TNF receptor leads to downstream signaling of pathways for inflammation, apoptosis and cellular regulation. The mechanism by which mutations in *TNFRSF1A* lead to uncontrolled inflammation in TRAPS is not entirely known. Proposed etiologies include decreased levels of circulating inhibitor soluble TNFR1, constitutive activation of the receptor, decreased TNF mediated apoptosis, and intracellular oxidative stress due to misfolding of the receptor in the endoplasmic reticulum [1].

## Case presentation

The patient presented at 7 months of age with seven days of fever, vomiting, diarrhea, and faint erythematous papular rash. She was born prematurely at 32 weeks and had delayed gross motor development compared to her fraternal twin. Laboratory tests showed c-reactive protein (CRP) 25.7 mg/dl, erythrocyte sedimentation rate (ESR) 50 mm, white blood cell count 19.6 10^3^/μL, hemoglobin 9.9 g/dL, and platelets 508 × 10^3^/μL. Liver enzymes and urinalysis were normal. Initial echocardiogram showed borderline dilated left anterior descending coronary artery (Z-score 2). Therefore, two doses of IVIG and aspirin were given for presumed incomplete Kawasaki Disease (KD). Though fever resolved with the second dose of IVIG, a dose of infliximab (10 mg/kg) was given due to borderline dilation of multiple coronary arteries on repeat echocardiogram (Z-scores 2.05–2.3). CRP improved to 3.4 mg/dl and she was discharged on low-dose aspirin.

Three days after discharge, the fever recurred to 103 °F without rash or other systemic symptoms. Laboratory testing again showed significant inflammation (CRP 26.2 mg/dl, ESR >140 mm). Urine culture grew *E.coli,* but she remained febrile despite appropriate intravenous antibiotics. Echocardiogram was normal. She subsequently developed diarrhea and abdominal distention, and abdominal CT showed a calcified appendicolith with regional ileus concerning for acute appendicitis. Appendectomy was deferred as she developed fever to 107 °F and hypotension requiring intensive care support. The fever persisted despite broadened antibiotic coverage.

**Fig. 1:**
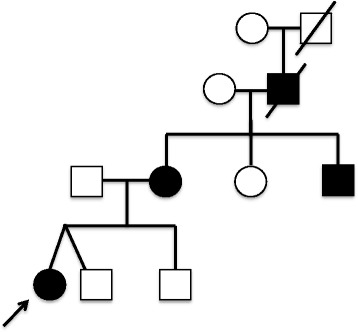
Family pedigree. *Arrow* indicates proband

Further investigation into our patient’s family history revealed that her mother and maternal uncle had previously been diagnosed with systemic juvenile arthritis due to recurrent attacks of fever, arthritis, rash, serositis, and conjunctivitis since infancy (Fig. 1). For them, several treatments were partially successful including etanercept and anakinra; infliximab caused severe flare of disease in the mother. By mother’s history, the patient’s maternal grandfather also suffered from similar attacks since childhood, but remained untreated and died at age 52 of complications of systemic amyloidosis.

**Fig. 2:**
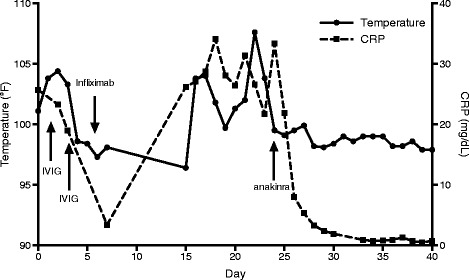
Daily maximum temperature curve superimposed with CRP values over the period from initial presentation to resolution of symptoms. Administration of therapies is indicated by *arrows*

Given this family history of chronic recurrent inflammatory disease, the diagnosis of TRAPS was considered and treatment with anakinra was initiated at 4 mg/kg/day. Her fever resolved within 48 h of initiating treatment. In addition, her abdominal distension improved and CRP normalized over two weeks (Fig. 2). Genetic testing (MNG Laboratories) confirmed a novel mutation at position 268A > C, causing the substitution of threonine to proline at amino acid 90, in the *TNFRSF1A* gene. At 15 months of age, the patient remains in remission on anakinra 2 mg/kg daily, with no evidence of subclinical inflammation. A trial of withdrawal of anakinra resulted in rapid elevation of serum inflammatory markers and return of fever. Gross motor development has also progressed to an age-appropriate level.

Affected and unaffected relatives (Fig. 1) were sequenced to query this mutation, and informed consent was obtained under an IRB approved protocol to Sanger sequence exon 3 of the *TNFRSF1A* gene (Additional file 1). Only the patient’s mother and maternal uncle were confirmed to share the same point mutation. No DNA was available from the deceased grandfather. Once an accurate diagnosis was established, the mother was treated with canakinumab with resolution of symptoms and normalization of inflammatory labs.

## Discussion and conclusions

Autoinflammatory diseases may present a diagnostic challenge to the pediatrician given their rarity, chronic recurrent nature of flares, and overlapping signs and symptoms with other disorders and amongst themselves. The increasing spectrum of autoinflammatory disorders has recently been reviewed [4–6]. For the clinician, a detailed history can provide invaluable information to differentiate between autoinflammatory disorders, as well as immunodeficiency or infection which also present in childhood, thereby reducing further delays in diagnosis and unnecessary diagnostic investigations. Extremes of hyperthermia may also be indicative of an underlying inflammatory condition, or condition affecting hypothalamic temperature regulation (i.e., malignant hyperthermia, spinal cord injury), rather than an infectious process [7–10]. Beyond the numeric temperature, recognition of characteristic patterns, such as duration of febrile attacks, and associated symptoms such as rashes, serositis, and arthritis, is often the key to diagnosing patients early [6]. A suspected diagnosis should be confirmed with genetic testing when possible. For our patient, a family history of a poorly defined inflammatory condition was crucial in making the correct diagnosis and initiating timely therapy. The mutation identified in this family, Thr90Pro in the *TNFRSF1A* gene, has not previously been reported in TRAPS.

This case demonstrates an unusual presentation of TRAPS in a young infant without the characteristic symptoms or history of recurrent fever episodes typically seen in older children and adults, and further emphasizes the importance of an accurate history in patients with fevers of unknown origin in order to pursue an appropriate clinical evaluation. Our patient had findings suggesting incomplete KD, urinary tract infection (UTI), and appendicitis, but these alternative diagnoses can be reconciled with the diagnosis of TRAPS. Transient coronary arteritis has been reported to occur in other autoinflammatory diseases [11, 12, 13]. Furthermore, the development of distinct autoinflammatory syndromes in patients with a history of KD has also been described [14] and it has been postulated that such immune dysregulation may also predispose patients to KD. The *E.coli* UTI may have served as the trigger for a TRAPS flare. Finally, sterile acute peritonitis is common in TRAPS and other autoinflammatory diseases and can mimic appendicitis such that one third of TRAPS patients undergo unnecessary appendectomy [1].

With the increasing availability of targeted biologic anti-cytokine treatments, new recommendations for the management of these unique patients have been proposed [15]. For TRAPS, treatment options include short-term glucocorticoids, with or without nonsteroidal anti-inflammatory drugs, anti-TNF therapy with etanercept, and IL-1 blockade, such as anakinra, as used in our patient. Notably, infliximab (Remicade), a monoclonal antibody directed against TNF-∝, has been shown to potentially trigger TRAPS flares [16], and its use is not recommended. Once on therapy, routine monitoring for subclinical inflammation as well as proteinuria, a manifestation of renal amyloidosis, is advised. As in this case, early diagnosis, treatment and close monitoring promote control of systemic inflammation, prevent long-term organ damage associated with systemic AA amyloidosis, and dramatically improve quality of life.

## Additional file

Additional file 1: This file contains the Sanger sequencing protocol. (DOCX 36 kb)

## Abbreviations

CRP: C-reactive protein
ESR: Erythrocyte sedimentation rate
KD: Kawasaki Disease
TRAPS: Tumor Necrosis Factor Receptor-Associated Periodic Syndrome
UTI: Urinary tract infection
