# Supplementary material for: Novel mutation identified in severe early-onset tumor necrosis factor receptor-associated periodic syndrome: a case report
Source: BMC Pediatr. 2017 Apr 20;17:108. doi: 10.1186/s12887-017-0856-2 (PMC5399385; doi:10.1186/s12887-017-0856-2)
Supplement: Additional file 1: — This file contains the Sanger sequencing protocol. (DOCX 36 kb) [file 12887_2017_856_MOESM1_ESM.docx]

**Additional file 1**

**Novel mutation identified in severe early-onset tumor necrosis factor receptor-associated periodic syndrome: A case report**

Suhas M. Radhakrishna, Amy Grimm, Lori Broderick, MD, PhD

Sanger Sequencing Protocol

Genomic DNA was isolated from whole peripheral blood using the Qiagen Gentra Puregene kit, following manufacturer’s instructions. Genomic DNA template (50ng) was used to amplify *TNFRSF1A* exon 3 using the following forward 5’-GGGTGCTGCTTCTTTCTCTG-3’ and reverse primer 5’- AGGGGAGAAGATGGGGTATG-3’ in 50ul reactions as previously described [1]. PCR reactions were submitted to Regeneron, Inc. (San Diego, CA, USA) for sequencing, and results aligned in Sequencher (Gene Codes Corp., Ann Arbor, MI, USA).

References

1. McDermott MF, Aksentijevich I, Galon J, et al. Germline mutations in the extracellular domains of the 55 kDa TNF receptor, TNFR1, define a family of dominantly inherited autoinflammatory syndromes. *Cell.* 1999 Apr 2;97(1):133-44.
